# Statistical Survey of Deaths from Nonmelanoma Skin Cancer in Japan during 54 Years

**DOI:** 10.1155/2011/293926

**Published:** 2011-01-16

**Authors:** Hisashi Ohtsuka

**Affiliations:** Department of Plastic and Reconstructive Surgery, Saiseikai Imabari 2nd Hospital, 1-7-43, Kita-hiyoshi-cho, Imabari-shi, Ehime 794-0054, Japan

## Abstract

The author analyzed the annual trends in the number of deaths from nonmelanoma skin cancer (NMSC) from 1955 to 2008 in Japan on the basis of the data from the Vital Statistics of Japan. The general trends in the number of deaths from NMSC were downward between 1979 to 1994, but upward after 1995. The general trends in age-standardized death rates were roughly downward, although the death rates plateaued after 1995. The recent annual increased ratio of deaths from NMSC was 3.8% (95% confidence interval: 2.7 *∼* 4.9%). The number and proportion of deaths from NMSC among the elderly were increasing in Japan. For females, more than 50% of the deaths occurred recently at or after 85 years of age, whereas, for males, this proportion was at or after 75 years of age, nearly reaching at or after 80 years of age.

## 1. Introduction

The incidence of nonmelanoma skin cancer (NMSC) has been increasing during the past half century in many countries [1–6]. Authors performed the analysis of changing trends in the number of deaths from NMSC in Japan from 1955 to 2000 [7]. This time, the author expanded the period of survey to 2008 and revised the analysis, using the new WHO standard population which is effective for the period 2000–2025 [8].

## 2. Material and Methods

The annual trends in the number of deaths from NMSC in Japan from 1955 to 2008, those in age-standardized death rates, those in 3-year moving average, those by 5-year age group, those by sex and age group, and those in the proportion of deaths by sex and age group were investigated on the basis of the data from the Vital Statistics of Japan, Statistics and Information Department, Minister's Secretariat, Ministry of Health, Labor and Welfare.

The corresponding revisions of the International Classification of Diseases (ICD) and codes were ICD-6 code 191 in 1955–1957, ICD-7 code 191 in 1958–1967, ICD-8 code 173 in 1968–1978, ICD-9 code 173 in 1979–1994, and ICD-10 code C44 in 1995–2008, respectively. The author, however, treated the results continuously from 1955 to 1967, because there was no difference between ICD-6 and ICD-7 concerning NMSC. The author treated the remaining revisions as discontinuous, because there was a minor difference between ICD-6/-7, ICD-8, ICD-9, and ICD-10. The author judged that it would be possible to obtain the general annual trends in the number of deaths from NMSC in Japan for 54 years, although, strictly speaking, there were four times minor changes of ICD classifications concerning NMSC between these periods.

The death rates were adjusted every year using the new WHO world standard population which is effective for the period 2000–2025. The 3-year moving averages of changes in the number of deaths were calculated in the periods of 1955–1967, 1968–1978, 1979–1994, and 1995–2008 in accordance with the following: {(*n*2 + *n*3 + *n*4)/3 − (*n*1 + *n*2 + *n*3)/3}/(*n*1 + *n*2 + *n*3)/3, where *n* indicated the number of deaths in each year. Thereafter, the annual increased ratio in each period was defined as the averages of the 3-year moving ones.

## 3. Results

**Figure 1:**
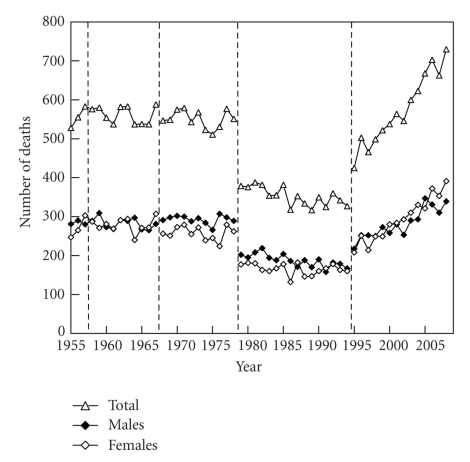
Annual trends in the number of deaths from nonmelanoma skin cancer (NMSC) in Japan (1955 ~ 2008), modified from Ohtsuka and Nagamatsu [7].

**Figure 2:**
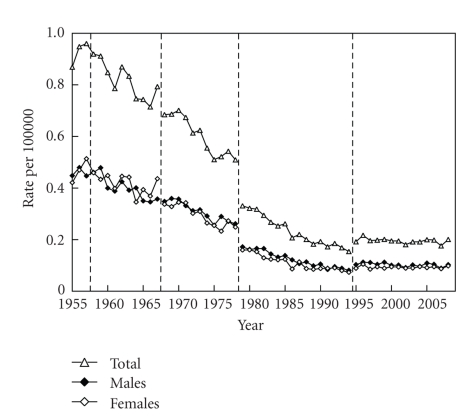
Annual trends in age-standardized death rates of NMSC in Japan (1955 ~ 2008), modified from Ohtsuka and Nagamatsu [7].

**Figure 3:**
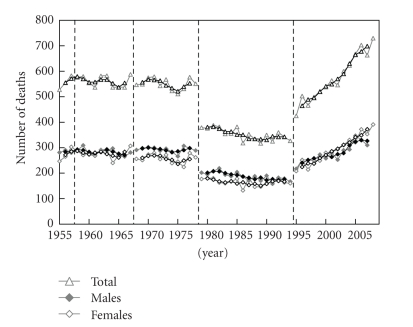
Annual trends in three-year moving average of NMSC in 1955–1967, 1968–1978, 1979–1994, and 1995–2008 in Japan, modified from Ohtsuka and Nagamatsu [7].

**Figure 4:**
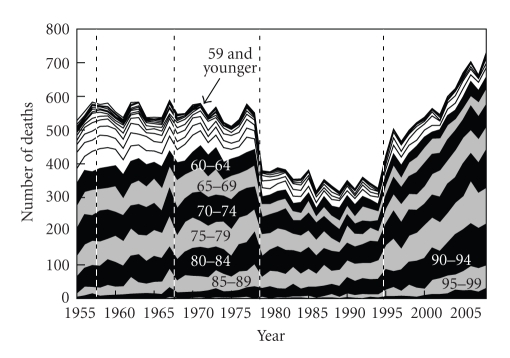
Annual trends in the number of deaths from NMSC by age group in Japan (1955 ~ 2008), modified from Ohtsuka and Nagamatsu [7].

**Figure 5:**
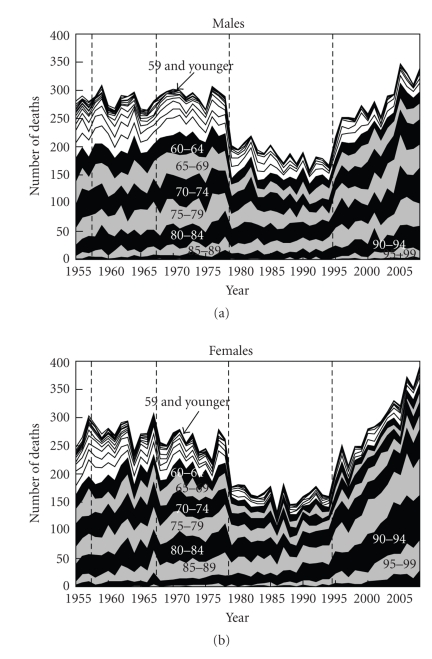
Annual trends in the number of deaths from NMSC by sex and age group in Japan (1955 ~ 2008), modified from Ohtsuka and Nagamatsu [7].

**Figure 6:**
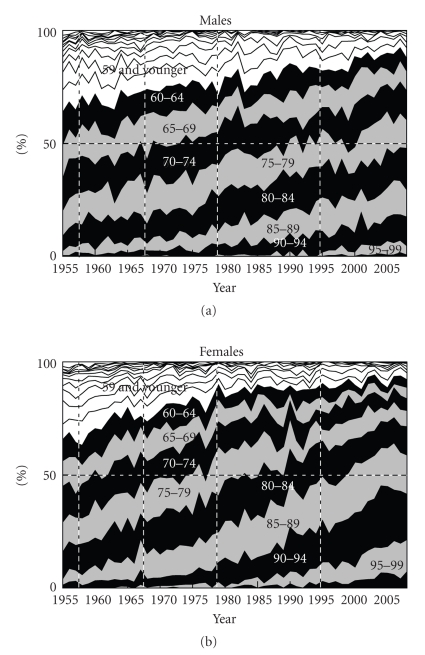
Annual trends in the proportion of deaths from NMSC by sex and age group in Japan (1955 ~ 2008), modified from Ohtsuka and Nagamatsu [7].

The general trends in the number of deaths from NMSC were downward between 1979 and 1994, but upward after 1995 (Figure 1). There was no gender difference in mortality. The general trends in age-standardized death rates were roughly downward, although the death rates plateaued after 1995 (Figure 2). The age-standardized death rate per 100,000 population in 2008 was 0.201 (0.103 in males, 0.098 in females). The annual increased ratio of deaths from NMSC from the 3-year moving increased ratio was 0.0% (95% confidence interval, CI: −1.4 ~ 1.4%) in 1955–1967, −0.1% (CI: −1.8 ~ 1.6%) in 1968–1978, −0.8% (CI: −2.0 ~ 0.4%) in 1979–1994 and 3.8% (CI: 2.7 ~ 4.9%) in 1995–2008, respectively (Figure 3). The ratio in males and females in each revision is shown in Table 1. The number and proportion of deaths among the elderly were increasing (Figures 4, 5, and 6). The peak age group of deaths was roughly the seventies in 1955–1979 and the eighties after 1980. However, there was a gender difference, as might be expected from the greater longevity of women. That is, there was a tendency of a sharper increase in the death ratio with age in females than in males (Figure 6). For females, more than 50% of the deaths occurred recently at or after 85 years of age, whereas, for males, this proportion was at or after 75 years of age, nearly reaching at or after 80 years of age.

## 4. Discussion

According to Jemal et al. [9], greater than 2 million cases of basal cell carcinoma (BCC) and squamous cell carcinoma (SCC) were expected to be newly diagnosed in the USA in 2010. Approximately 80% of NMSCs are also said to be BCCs and 20% SCCs [2, 10]. Stratton [11] described that NMSC received a disproportionate share of attention because mortality was relatively low. Cutaneous SCCs are, however, associated with a substantial risk of metastasis, unlike almost all BCCs [2]. Consequently, SCC is considered to be the main cause of death in NMSCs. The population-based case-fatality rates for BCC and SCC were estimated to be roughly 0.05% and 0.7%, respectively by Weinstock in 1997 in the USA [12]. Nolan et al. [3] described that most fatalities from NMSC in Western Australia were caused by 1 of 3 neoplasms: SCC, Merkel cell carcinoma, and adenosquamous carcinoma, and that the mortality rate for NMSC was 0.8% under the stringent criteria of cancer-related mortality except for cancers of the lip.

In 2000, Salasche [13] estimated that there were approximately 200,000 new SCCs each year in the USA and between 1,300 and 2,300 people died each year as a result of NMSC, mostly metastatic SCC. In Australia, there were nearly 400 deaths from NMSC annually [14]. And, the majority of NMSC deaths were due to SCCs, had primary sites associated with significant sun exposure, and occurred in older men [14]. There is no estimated incidence of NMSC yet in Japan, although Ishihara [15] estimated 5,000 BCC and 2,000–2,500 new SCC cases/year in 1994. The author could not believe those figures which were estimated from a survey in 75 university hospitals and 25 large hospitals. The author estimates that many NMSCs were excluded from the statistics, because many of them were treated at smaller hospitals or even at clinics. At least, a tenth to a fifth of 2 million NMSC cases/year/314.7 million population in 2010 in the USA, that is, 200,000–400,000 new NMSC cases/year/127.6 million population are estimated in 2010 in Japan in due consideration of the difference between races and/or between geographic distributions in the USA and Japan.

The development of surgical procedures may have a close relationship with the decrease in the number of deaths from NMSC after 1979 in Japan, in addition to minor changes of ICD classification. Many typical NMSCs developed in injured or chronically diseased skin, including skin affected by long-standing ulcers and radiation dermatitis, could from then on be treated completely by wide excisions, followed by appropriate reconstructive procedures such as newly developed free flaps and pedicled or free musculocutaneous flaps in the 1970s [16, 17], resulting in a remarked reduction in the number of deaths from NMSC in Japan [7]. The new increase after 1995 in Japan may be explained firstly by the increasing proportion of elderly subjects with an actinic keratosis- (AK-) induced SCC in poor condition, secondly by improvements in diagnosis, and thirdly by the greater precision in reporting on death certificates from any cause as associated with NMSC [7].

In Japan, the cause of death has been established from death certificate. And, death certificates have been forwarded through city or town office to Statistics and Information Department of Ministry of Health, Labor and Welfare. The validity and reliability of the data have been believed to be sufficiently trustworthy because the proportion of histopathological diagnosis especially in malignant tumors has increased remarkably year by year. The cause of death has been analyzed every year since 1899 and has been used for the analyses of many malignant tumors, according to WHO ICD classification. Stang and Jöckel [18] suggested that a limitation of the NMSC mortality study results was the potential inaccuracy of routine universal cause-of-death certification. The validity of cause-of-death certification had not been studied for NMSC in Germany. 

Nevertheless, statistics based on routine death certification may provide a rough guide to the magnitude of NMSC mortality. Girschik et al. [14] also stated that overall their study found that misclassification of skin cancer deaths in Western Australia Cancer Registry was minimal with less than 1% of cases miscoded.

The major strength of this paper is the large sample size from which the data is drawn. In addition, it gives a very recent analysis of the death rate from NMSC in Japan. On the other hand, there still remains a limitation of the validity and reliability of the cause of death in NMSC, in spite of increasing ratio of histopathological diagnosis. Therefore, retrospective study for the correlation between the cause of death and histopathological diagnosis should sometimes be performed to decrease the proportion of misclassification or histopathological misdiagnosis of skin cancer deaths by re-examinations of histological sections.

In addition, another problem prohibiting from accuracy of coding of the cause of death apart from histopathological diagnosis might have arisen, or may gradually arise. The cause of death may be misdiagnosed or miscoded as pneumonia, chronic lymphoid leukemia, death of old age with dementia, and others, in spite of existence of invasive and metastatic SCC, when the elderly may die at nursing homes or even hospitals without biopsy or autopsy, and medical personnel may be less familiar with NMSC, resulting in the decreased number of death from NMSC. Hasuo et al. [19] described that the validation of certified diagnosis was less reliable in the aged population.

The population of Japan has not increased remarkably in the last 30 years and has been rather plateaued to slightly decreased in the last 10 years. However, proportion of the elderly of 65 years and over has increased year by year, reaching 23.1% (males: 20.3%, females: 25.8%) of total population in 2010. Life expectancy at birth for females in 2010 was 86.44 years, which was the first rank in the world, and that for males was 79.59 years, the fifth rank, according to the Ministry of Health, Labor and Welfare in Japan.

The most important prevention via routine screening and better treatment of the elderly can help identify potential lethal lesions, improve prognosis, and reduce deaths. Although there may always be the occasional very elderly patient with multiple comorbid medical conditions whose skin cancer can be more easily handled with simple electrodesiccation and curettage, Mohs surgery does appear to be a safe and effective therapy for the great majority of very elderly patients in the USA [20, 21].

## 5. Conclusions

The author rightly emphasized based on his data analysis that the elderly had increased risk of death from NMSC. In recent years, more than 50% of the deaths among females occurred at or after 85 years of age and the corresponding age for males was at or after 75 years, nearly reaching at or after 80 years of age. The most important prevention via routine screening and better treatment of the elderly can help identify potential lethal lesions, improve prognosis, and reduce deaths.

## Figures and Tables

**Table 1:** The annual increased ratio of deaths from nonmelanoma skin cancer (NMSC) (% and 95% confidence interval) from 3-year moving average by sex in each ICD revision in Japan.

Revision (year)	Males	Females	Total
ICD-6,7 (1955 ~ 1967)	−0.4 (−1.9 ~ 1.0)	0.5 (−2.0 ~ 2.9)	0.0 (−1.4 ~ 1.4)
ICD-8 (1968 ~ 1978)	0.1 (−1.2 ~ 1.3)	−0.2 (−2.9 ~ 2.6)	−0.1 (−1.8 ~ 1.6)
ICD-9 (1979 ~ 1994)	−1.0 (−2.5 ~ 0.5)	−0.5 (−2.8 ~ 1.9)	−0.8 (−2.0 ~ 0.4)
ICD-10 (1995 ~ 2008)	2.9 (0.7 ~ 5.1)	4.7 (3.1 ~ 6.3)	3.8 (2.7 ~ 4.9)
